# Diagnostic Errors in Sepsis: A Systematic Review of the Literature

**DOI:** 10.3390/jcm15208152

**Published:** 2026-10-20

**Authors:** Chun Shing Kwok, Soyoung Lee, Azreen Afzal, Sadie Bennett, Jaime Halvorsen, Clare Hammell

**Affiliations:** 1 Department of Cardiology, Mid Cheshire Hospitals NHS Foundation Trust, Crewe CW1 4QJ, UK; afzal.azreen@mcht.nhs.uk; 2 Department of General Surgery, Jersey General Hospital, Jersey JE1 3QS, UK; soyoung314@gmail.com; 3 Department of Cardiology, University Hospitals of North Midlands NHS Trust, Stoke-on-Trent ST4 6QG, UK; sadie.bennett@uhnm.nhs.uk; 4 Cardiovascular Clinical Research Facility, University of Oxford, Oxford OX3 9DU, UK; 5 Mid Cheshire Hospitals NHS Foundation Trust, Crewe CW1 4QJ, UK; jaime.halvorsen@mcht.nhs.uk (J.H.); clare.hammell@mcht.nhs.uk (C.H.)

**Keywords:** sepsis, diagnostic error, misdiagnosis, mortality, complications

## Abstract

**Background/Objectives**: Sepsis is a major cause of hospitalization and mortality which may be prevented with early diagnosis. This systematic review aims to evaluate the literature on diagnostic errors in sepsis. **Methods**: We conducted a systematic review to evaluate the rate and outcomes of diagnostic errors in sepsis. We searched MEDLINE and EMBASE in September 2026 for studies that evaluated diagnostic errors in sepsis. Data were extracted into tables and narratively synthesized. **Results**: Twenty-six studies involving 868,057 patients diagnosed with sepsis were included. Definitions of diagnostic error varied and included misdiagnosis, missed diagnosis, delayed diagnosis, failure of recognition systems, and overdiagnosis. Among adults, reported rates of diagnostic error ranged from 0.6% to 86.2%, depending on the definition used. Misdiagnosis of infection source occurred in 11.6% of patients, sepsis misdiagnosis rates ranged from 8.3% to 25.3% in most cohorts, and delayed recognition ranged from 20.0% to 72.9%. In pediatric studies, missed or delayed sepsis occurred in 2.9% to 16.9% of patients. Diagnostic error was associated with delayed antibiotic administration, increased illness severity, longer hospitalization, and higher intensive care utilization. However, findings regarding mortality were inconsistent, with several studies reporting no significant association after adjustment. In children, missed sepsis was associated with increased risk of intensive care admission, but no difference in mortality. **Conclusions**: Diagnostic error in sepsis is common and variably defined across studies. Evidence linking diagnostic error to mortality and intensive care admission is inconsistent; however, diagnostic error is frequently associated with delayed treatment.

## 1. Introduction

Sepsis refers to a systemic illness caused by microbial invasion of normally sterile parts of the body [1]. It is defined as life-threatening organ dysfunction caused by a dysregulated host response to infection, where organ dysfunction can be identified as an acute change in total Sequential Organ Failure Assessment (SOFA) score of 2 or more points [2]. For children, the Society of Critical Care Medicine task force recommends that sepsis in children be identified by a Phoenix Sepsis Score of at least 2 points in children with suspected infection, which indicates potentially life-threatening dysfunction of the respiratory, cardiovascular, coagulation, and/or neurological systems [3]. It is a condition that can progress rapidly, and in severe cases, one or more organ systems fail, which can result in hypotension from septic shock [4]. In the United States, the incidence of sepsis hospitalizations is estimated to range between 28 and 121 per 1000 hospitalizations depending on the coding strategy used [5]. It is important, as the average mortality reported by a review of 170 studies was 24.4% at 30 days [6]. In the United Kingdom in 2016 to 2017, an analysis of 133 hospital trusts had 77,996 admissions for sepsis and 15,851 deaths [7]. Globally, sepsis accounts for 49 million cases each year, resulting in deaths reaching 11 million [8]. The World Health Organization (WHO) reported that the deaths could be prevented through early diagnosis and appropriate clinical management [9]. The key recommendations from the Surviving Sepsis Campaign 2021 guidelines emphasize the importance of early recognition and screening, rapid antimicrobial therapy, fluid resuscitation, vasopressor therapy, lactate measurement, source control, corticosteroids, mechanical ventilation, ICU care, and post-discharge care [10].

Diagnostic error is a broad and non-specific concept that warrants careful consideration in the context of sepsis. It encompasses misdiagnosis or delayed diagnosis, where an initial diagnosis is subsequently revised to a different diagnosis [11], as well as delayed recognition, where patients may be triaged as low priority [12]. Distinguishing diagnostic error from true disease progression can be challenging, particularly because demonstrating progression requires high-quality clinical data from both the initial and subsequent evaluations. In sepsis, misclassification of the site of infection may also be regarded as a form of misdiagnosis [13]. More broadly, diagnostic accuracy can be conceptualized using the framework of true-positive, true-negative, false-positive, and false-negative diagnoses. Within this framework, false-positive diagnoses represent overdiagnosis, whereas false-negative diagnoses represent underdiagnosis [14]. Diagnostic errors in sepsis may also relate to missed sepsis, which could be evaluated by emergency department visits that were followed up for readmissions with sepsis and those where patients who are admitted with sepsis were referred for previous encounters in the emergency department [15,16].

We conducted a systematic review of the literature to evaluate diagnostic errors in sepsis. Our objective was to determine how diagnostic errors related to sepsis have been defined in the literature, how frequently they occur, and whether there are differences in outcomes associated with misdiagnosis.

## 2. Materials and Methods

This review was prepared in accordance with the recommendations of the PRISMA 2020 criteria [17]. The PRISMA 2020 Checklist is provided in Supplementary Material. Although the review was not registered, a protocol was developed and followed.

### 2.1. Eligibility Criteria

We selected studies that evaluated diagnostic errors in patients with sepsis. There was no restriction on the definition of diagnostic error, which could include missed sepsis, misdiagnosis of sepsis, and delayed recognition of sepsis. For a study to be included, it had to have a definition for diagnostic error or misdiagnosis of sepsis and evaluate one or more of the following: (i) the number of patients who had a misdiagnosis of sepsis and another set of patients who were not misdiagnosed, or (ii) outcomes for patients with a misdiagnosis of sepsis compared to patients who were correctly diagnosed. There was no restriction on the language of the study, and we used Microsoft Translator to translate published articles in other languages when texts were not in English. However, we only included studies or reviews with multiple patients and original data, so editorials, comments, and case reports were excluded.

### 2.2. Search Strategy

We used the OVID platform (Wolters Kluwer, New York, NY, USA) to search MEDLINE and EMBASE on 24 September 2026 with no limits on date or language. The exact search terms were: (missed sepsis or (missed diagnos* adj3 sepsis) or (unrecogni* adj1 sepsis) or (misdiagnosis and sepsis) or (missed diagnosis and sepsis) or missed septicemia or (missed diagnos* adj3 septicemia) or (unrecogni* adj1 septicemia) or (misdiagnosis and septicemia) or (missed diagnosis and septicemia) or missed septicaemia or (missed diagnos* adj3 septicaemia) or (unrecogni* adj1 septicaemia) or (misdiagnosis and septicaemia) or (missed diagnosis and septicaemia)) or ((diagnostic error adj3 sepsis) or (diagnostic error adj3 septicaemia) or (diagnostic error adj3 septicemia) or (delayed diagnosis adj3 sepsis) or (delayed diagnosis adj3 septicaemia) or (delayed diagnosis adj3 septicemia) or (failure to diagnose adj3 sepsis) or (failure to diagnose adj3 septicaemia) or (failure to diagnose adj3 septicemia) or (underdiagnosis adj3 sepsis) or (underdiagnosis adj3 septicaemia) or (underdiagnosis adj3 septicemia) or (overdiagnosis adj3 sepsis) or (overdiagnosis adj3 septicaemia) or (overdiagnosis adj3 septicemia) or (recognition adj3 sepsis) or (recognition adj3 septicaemia) or (recognition adj3 septicemia)).

The bibliographies of included and potentially relevant studies were reviewed for additional studies that met the inclusion criteria.

### 2.3. Selection Process and Data Collection Process

Two reviewers (CSK and SL) independently screened all titles and abstracts from the search for studies that met the inclusion criteria. The studies that potentially met the inclusion criteria were retrieved and reviewed for final inclusion. The decision to include or exclude studies was made by consensus, and data extraction was carried out independently by two reviewers (CSK and SL). The data collected included study design, country of study origin, year, sample size, mean age, percentage of the cohort that was male, inclusion criteria, definition of diagnostic error in sepsis, setting where the study took place, and results. Results were defined by the number of cases of diagnostic error in sepsis and outcomes associated with diagnostic error in sepsis.

### 2.4. Study Risk of Bias Assessment

The methodological quality assessment for the included studies was conducted based on some of the relevant elements from the Ottawa-Newcastle scale for non-randomized studies [18]. The risk of bias was independently extracted by two reviewers (CSK and SL). Disagreements were resolved by auditing the data by SL. The criteria in the quality assessment were: (i) Was the cohort representative of typical patients with sepsis? (ii) Does the study have a reliable diagnostic error evaluation? (iii) Does the study evaluate an outcome that was not present at the start of the study? (iv) Is there use of adjustments if appropriate? (v) Is there a reliable evaluation of the outcome? (vi) Is the follow-up period for outcomes appropriate? and (vii) Is the follow-up adequate (<5% missing or lost data)?

### 2.5. Synthesis Methods

Data were extracted into pre-designed and piloted tables in Microsoft Word (Microsoft 365). The significant heterogeneity in study methodology meant that we could not perform meta-analysis, so the study findings were narratively synthesized. The definition, rate, and outcomes associated with diagnostic error were determined. A subgroup evaluation was performed for studies of children.

## 3. Results

### 3.1. Description of Studies

**Figure 1:**
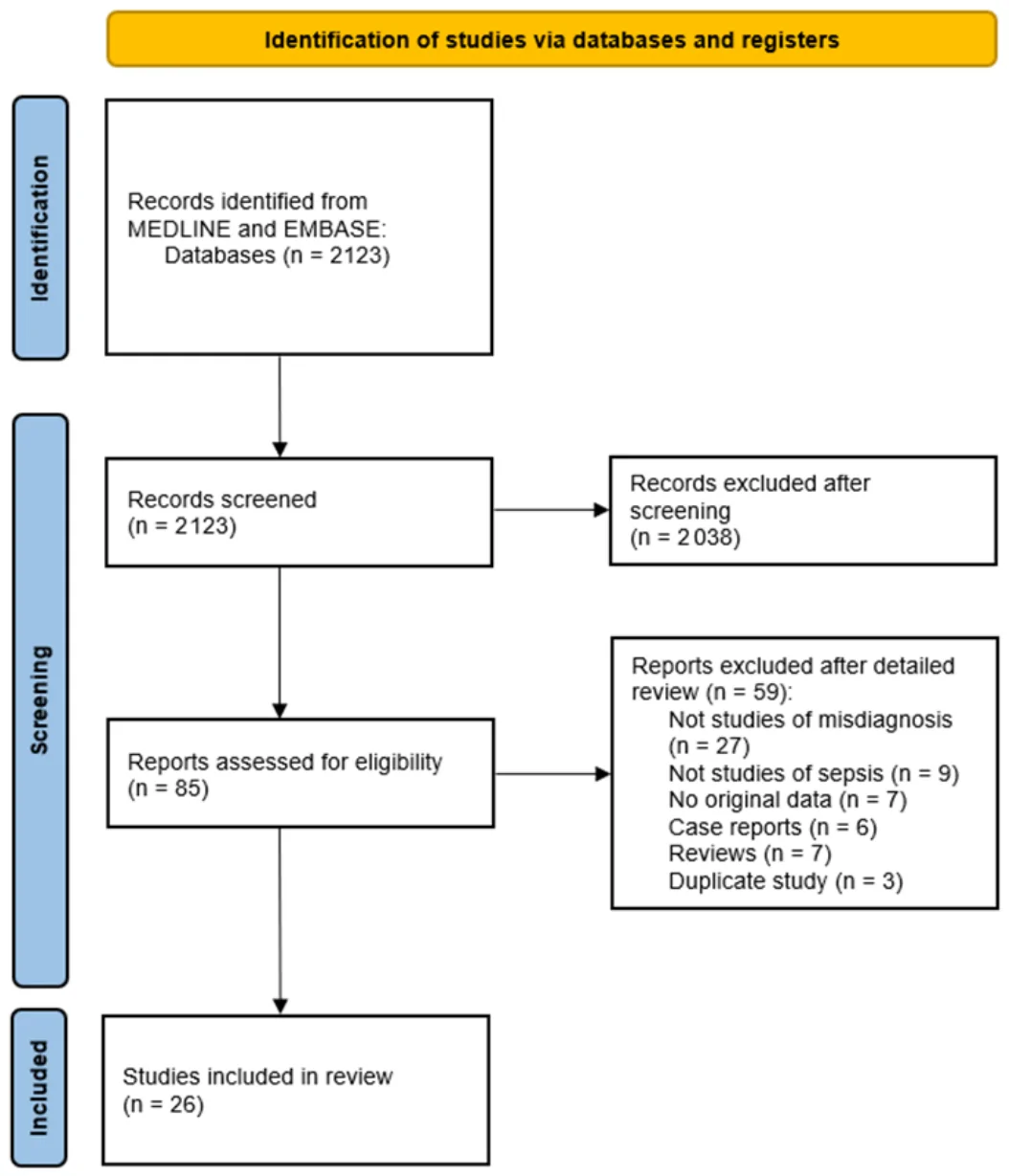
Flow diagram of study selection.

A total of 26 studies were included in this review, and the process of study selection is shown in Figure 1 [11,12,13,14,15,16,19,20,21,22,23,24,25,26,27,28,29,30,31,32,33,34,35,36,37,38].

Table 1 shows the description of the included studies. There were 23 retrospective cohort studies, 2 studies that were secondary analyses of data collected for other studies, and one prospective cohort study. These studies took place in the United States, Japan, Italy, Hungary, Germany, the Netherlands, China, and Australia, and they took place between 2004 and 2024. Excluding the patient contribution from 3 studies [15,16,29] because of possible overlap with other studies, there were a total of 873,140 patients included in the analysis with 868,057 diagnosed with sepsis, 2451 with infections or suspected infections, 2386 without a diagnosis of sepsis and 246 with a diagnosis of heart failure. Five of the studies reviewed were cohorts of children [11,15,34,35,36], while the remaining 21 studies evaluated misdiagnosis in adult patients. The average proportion of patients who were male that was reported in 16 studies was 54.8%.

### 3.2. Study Quality Assessment

The study quality assessment is shown in Table 2. There were 25 studies with cohorts that were representative of patients with sepsis. The study by Horberg et al. was a cohort of patients who were seen in the emergency department with fluid and electrolyte disorders and altered mental status [16]. Another evaluation only included patients with surgical mortality, with a subgroup having a diagnosis of sepsis [33]. Methods for diagnostic error were considered to be satisfactory for all but one study, which focused on patients who only had acute decompensated heart failure if they did not have sepsis [31]. There were 10 studies that evaluated outcomes, and the most common was in-hospital or 30-day mortality. Only 3 of the studies used adjustments in the outcome analysis [13,21,23]. Among studies that evaluated outcomes, all 10 had reliable outcome ascertainment and reasonable duration for follow-up. Missing data was significant in 6 studies [13,21,22,28,30,32] and not significant in 2 studies [12,31]. For 3 studies, missing data were unclear [23,35,36], and for 15 studies there were no outcomes.

### 3.3. Rate of Diagnostic Error in Sepsis in Adults

**Figure 2:**
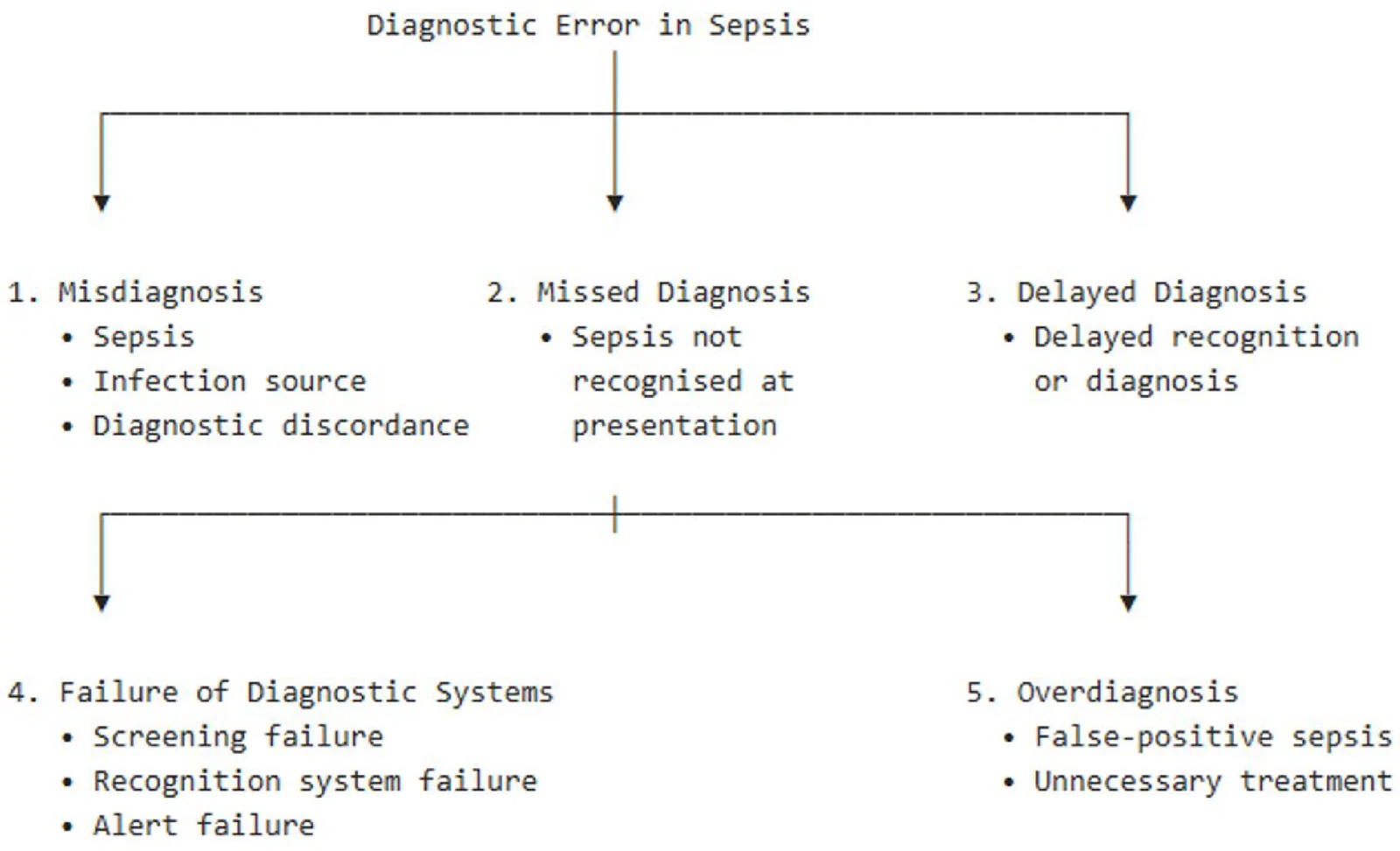
Key domains in diagnostic error in sepsis.

The definitions and rate of diagnostic error in sepsis are shown in Table 3. Considerable heterogeneity was observed in the definitions of diagnostic error across studies. Definitions were broadly categorized into five domains: (1) misdiagnosis of sepsis or the site of infection, including discordance between initial and final diagnoses; (2) missed diagnosis, characterized by failure to recognize sepsis at presentation; (3) delayed diagnosis or delayed recognition of sepsis; (4) failure of diagnostic screening or recognition systems to identify patients with sepsis; and (5) overdiagnosis, including false-positive sepsis diagnoses (Figure 2).

Among adult populations, reported rates of diagnostic error in sepsis varied substantially depending on the definition used. Misdiagnosis, missed diagnosis, delayed diagnosis, and failure of recognition ranged from 0.57% to 86.2% [12,13,14,16,19,20,21,22,23,24,25,26,27,28,29,30,31,32,33,34,38,39]. Misdiagnosis of the site of infection occurred in approximately 11.6% of patients [23], while sepsis misdiagnosis rates ranged from 8.3% to 25.3% in most cohorts, although rates as high as 77.8% were reported among patients requiring ICU escalation [26]. Delayed recognition of sepsis ranged from 20.0% to 34.3% when defined by delays exceeding 6 to 12 h and reached 72.9% when sepsis was omitted from the provisional diagnosis [19]. Failure to recognize sepsis was also common, occurring in 33.0% of emergency department presentations [12] and 86.2% of cases assessed by emergency medical services personnel [38]. Studies evaluating diagnostic test performance reported false-positive rates of 11.0%, false-negative rates of 78.7%, and sepsis overdiagnosis rates of 15.1% [14,30].

### 3.4. Rate of Diagnostic Error in Sepsis in Children

Among pediatric populations, missed or delayed diagnosis of sepsis remained common, although rates were generally lower than those reported in adult cohorts. Missed sepsis was reported in 2.9% (32/1094) to 8.3% (15,331/184,157) of children with sepsis [34,35], while delayed diagnosis occurred in 16.9% (109/646) of pediatric sepsis cases [11]. Additional studies reported missed sepsis among 8.1% (158/1945) of hospital admissions with sepsis [15] and 5.5% (53/972) of children presenting to the emergency department [36]. Overall, estimates of missed or delayed pediatric sepsis ranged from 2.9% to 16.9%.

### 3.5. Harm or Potential Impact on Outcomes for Patients with Misdiagnosis of Sepsis

Table 4 shows that ten studies of adults [12,13,14,20,21,23,28,29,30,32] and two studies of children [35,36] evaluated the association between diagnostic error and clinical outcomes. In one study, in-hospital mortality was higher among patients with an incorrect source diagnosis than among those with a correct diagnosis (24.8% vs. 13.7%, *p* < 0.01), with misdiagnosis independently associated with mortality after adjustment (aOR 2.66, 95% CI 1.45–4.89) and in a propensity-matched analysis (aOR 3.03, 95% CI 1.24–7.38) [13]. The same study reported a higher 28-day mortality among patients with source misdiagnosis (20.6% vs. 13.4%, *p* = 0.05), although length of stay did not differ significantly [13]. In another study, patients with late recognition had higher illness severity scores (SOFA 9.2 ± 1.6 vs. 6.5 ± 1.3, *p* < 0.001), longer hospital stays (14.6 ± 2.5 vs. 12.5 ± 1.2 days, *p* < 0.001), and higher mortality (25.5% vs. 10.3%, *p* = 0.010) compared with those recognized early [22]. Also, patients with missed sepsis were less likely to receive timely antibiotics within 60 min than those with recognized sepsis, while persistent missed sepsis was associated with particularly low rates of prompt treatment (52.2% vs. 72.6%) [30]. However, this study did not demonstrate significant differences in mortality.

The findings regarding unrecognized sepsis in adults were inconsistent. One large emergency department study found lower crude mortality among patients with unrecognized sepsis compared with recognized sepsis (2.3% vs. 10.4%), although unrecognized patients experienced longer emergency department stays [12]. Several studies found no clear association between diagnostic error and mortality or intensive care admission after adjustment [28,32]. One study reported no significant association between misdiagnosis and 30-day mortality (aOR 0.8, 95% CI 0.3–1.9), ICU admission (aOR 1.3, 95% CI 0.6–3.0), or duration of hospitalization, although misdiagnosed patients were more likely to receive broad-spectrum antibiotics (aOR 4.0, 95% CI 1.8–8.8) [23].

In pediatric studies, missed sepsis was associated with delayed antibiotic administration and greater likelihood of subsequent intensive care admission. Children with missed sepsis were nearly three times more likely to require ICU admission within 24 h (87.5% vs. 30.5%) and were less likely to receive intravenous antibiotics within 90 min of emergency department triage (22.6% vs. 55.1%; RR 0.4, 95% CI 0.3–0.7) [35,36]. No significant difference in 30-day mortality was observed (0% vs. 1.4%) [35].

## 4. Discussion

Rates of diagnostic error in sepsis vary widely across populations, but several patterns emerge. In adults, misdiagnosis varies from one in four to one in ten patients depending on the definition used, while delayed recognition of sepsis occurs in one in five to three out of four adult patients. In pediatric studies, missed or delayed sepsis occurred in 2.9% to 16.9% of patients. Together, these factors highlight that the relationship between errors in diagnosis of sepsis and outcomes is not straightforward and may reflect underlying differences in case severity and clinical context rather than the direct consequences of diagnostic error.

Patient pathways provide a useful lens through which to identify potential missed opportunities in sepsis care, as they map the sequence of clinically relevant events from a patient’s first contact with healthcare services through subsequent assessment, diagnosis, treatment, and follow-up [39]. Although there is no single universally accepted diagnostic pathway for sepsis, a sequence of recognition, assessment, diagnosis, treatment, and reassessment is consistent with the principles of the Surviving Sepsis Campaign [10]. Diagnostic errors may occur at any stage of this pathway and should be interpreted within the wider context of an individual patient’s clinical trajectory.

Sepsis pathways are highly heterogeneous. Some patients present early in the community with non-specific symptoms and physiological stability, making sepsis difficult to recognize, while others present later with more overt manifestations of infection but are at greater risk of adverse outcomes because of disease progression. Patients may also initially present for unrelated reasons and subsequently develop healthcare-associated infections that progress to sepsis. Such variability can make it difficult to distinguish true diagnostic error from the natural evolution of illness.

Recognition, assessment, and diagnostic decision-making are particularly vulnerable to error because the clinical presentation of sepsis is often heterogeneous and may overlap with alternative diagnoses, especially during the early stages of illness. Furthermore, the first healthcare professional to assess a patient may vary according to the healthcare setting and may include paramedics, nurses, primary care physicians, emergency department clinicians, or hospital specialists. Differences in training, experience, and exposure to severe infection may influence clinical judgment and contribute to variation in diagnostic accuracy.

Ongoing monitoring and reassessment are therefore critical components of the diagnostic process. Not all patients initially present with sepsis; some present with uncomplicated infection and subsequently deteriorate as organ dysfunction develops. Reassessment provides an opportunity to recognize evolving sepsis, refine the working diagnosis, and identify patients whose condition is not responding as expected. Repeated clinical evaluation is also necessary to confirm that the source of infection has been correctly identified and that antimicrobial therapy, source-control measures, fluid resuscitation, and other supportive treatments remain appropriate. This highlights that sepsis diagnosis should be viewed as a dynamic and iterative process rather than a single-point clinical decision.

Importantly, retrospective reviews frequently identify earlier encounters as potential “missed opportunities”; however, such assessments may not adequately consider whether sepsis was reasonably diagnosable at that specific point in time. The most clinically meaningful missed opportunities are those in which earlier recognition, escalation, treatment, or safer discharge decisions could realistically have altered patient outcomes or reduced healthcare resource utilization. Further research, particularly prospective studies examining patient pathways in real time, is needed to determine when earlier intervention is both feasible and clinically justified and to better distinguish avoidable diagnostic errors from the inherent uncertainty associated with the progression of infectious illness.

The clinical implications of diagnostic errors can be substantial given the time-sensitive nature of severe infections. Misdiagnosis of sepsis may contribute to both underdiagnosis and overdiagnosis. Underdiagnosis can result in delays to appropriate treatment, leading to disease progression and, in severe cases, adverse outcomes such as long-term complications or death. Although prompt and optimal treatment improves outcomes, delayed recognition may reduce the effectiveness of subsequent interventions.

Conversely, overdiagnosis may lead to unnecessary antibiotic exposure and inappropriate use of broad-spectrum antimicrobial agents. This may contribute to the development of antimicrobial resistance, which remains a major public health concern. Antibiotics are also associated with potential adverse effects, including *Clostridioides difficile* infection and other drug-related complications, exposing patients to avoidable risks when treatment is not clinically indicated.

The consequences of antimicrobial overuse may extend beyond the immediate episode of care. Patients may subsequently develop infections caused by more resistant organisms, which can increase the risk of treatment failure, recurrent infections, and hospital readmissions. These considerations highlight the importance of antimicrobial stewardship and careful diagnostic assessment when initiating broad-spectrum antibiotic therapy, while recognizing the need for individualized clinical decision-making based on the patient’s presentation and risk profile. Antimicrobial stewardship programs are designed to optimize antibiotic use and have been associated with reduced incidence of infections and colonization with antibiotic-resistant bacteria, as well as improved patient outcomes and fewer adverse events [40].

In this review, most of the evidence was from high-income health systems, particularly the United States, with only a few studies considering community settings. The setting in which each study was conducted remains an important consideration, as the likelihood and nature of diagnostic error differ substantially between community general practice, emergency departments, inpatient wards, and intensive care units. Diagnostic resources and access to investigations are often more limited in community and emergency settings, making it unsurprising that some patients initially receive an alternative diagnosis before sepsis becomes apparent. In contrast, hospital wards and intensive care units typically have greater diagnostic capacity and more continuous monitoring, which may support earlier recognition. These contextual differences highlight that variation in diagnostic error rates may reflect differences in system capability as much as clinician performance, and they underscore the importance of interpreting misdiagnosis data within the clinical environment in which it arises.

This review demonstrates that diagnostic error in sepsis is a heterogeneous construct encompassing multiple distinct phenomena. The identified definitions can be broadly classified into five domains: (1) misdiagnosis of sepsis or the site of infection, including discordance between initial and final diagnoses; (2) missed diagnosis, defined as failure to recognize sepsis at presentation; (3) delayed diagnosis or delayed recognition of sepsis; (4) failure of diagnostic screening or recognition systems to identify patients with sepsis; and (5) overdiagnosis, including false-positive sepsis diagnoses. Although these domains are frequently considered collectively under the term diagnostic error, greater precision in terminology is warranted. Future studies should explicitly distinguish between these domains to improve consistency in measurement, interpretation, and reporting.

This review has several important limitations. First, substantial inconsistencies in how studies defined diagnostic errors in sepsis and considerable methodological heterogeneity prevented any form of meta-analysis. Second, aside from one secondary analysis of a prospective cohort study, all included studies were retrospective and observational, making them vulnerable to confounding and various forms of bias. In particular, studies using look-back designs are susceptible to immortal time bias and survivor bias, as patients who die before re-presenting with sepsis are not captured in administrative datasets, likely underestimating the true harm of errors in diagnoses [41]. Additionally, many of the included studies were available only as conference abstracts, limiting methodological appraisal and potentially introducing publication bias toward more striking misdiagnosis rates. None of the included studies reported outcomes stratified by sex or ethnicity, which is a key gap given emerging evidence of demographic disparities in sepsis recognition and time to treatment [42]. Third, we identified limited studies examining misdiagnosis of sepsis in community settings, despite general practice often being the first point of contact for patients with infection. Finally, we found no studies that directly addressed strategies to reduce diagnostic error in sepsis, highlighting a critical need for research focused on improving diagnostic accuracy and enhancing the quality of care for patients with sepsis.

## 5. Conclusions

In conclusion, diagnostic errors in sepsis are relatively common, though reported rates vary widely due to inconsistent definitions and study methods. Despite this, errors related to the diagnosis of sepsis were not consistently associated with higher mortality or increased ICU use, suggesting that its clinical impact may be more nuanced than expected. Continued, methodologically robust research is needed to determine when errors in sepsis diagnosis may lead to preventable harm and how diagnostic accuracy can be improved.

## Figures and Tables

**Table 1:** Study design, patient characteristics, and participant inclusion criteria of the included studies.

Study ID	Study Design; Country; Year	No. of Patients	Mean Age	% Male	Participant Inclusion Criteria
Abe 2019 [13]	Secondary analysis of a prospective cohort study; Japan; 2017 to 2018.	974 infections	Median 78	60.4%	Patients were adults with suspected infection in the Japanese Association for Acute Medicine Sepsis Prognostication in Intensive Care Unit and Emergency Room study.
Barker 2023 [19]	Retrospective cohort study; Australia; 2018 to 2020.	59 sepsis	74.3	71.2%	Patients were adults admitted acutely to seven public hospitals in Queensland, Australia.
Bosetti 2024 [12]	Retrospective cohort study; Italy; 2022.	11,508 sepsis	76.1	54.9%	Patients were adults admitted to an ED in the Lombardy region of Italy with a diagnosis of sepsis.
Cifra 2021 [15]	Retrospective cohort study; USA; 2010 to 2011.	1945 severe sepsis/septic shock	8.2	52.9%	Patients were children admitted with severe sepsis/septic shock in the ED.
Hamdon 2019 [20]	Retrospective cohort study; USA; Unclear.	22 sepsis	-	-	Patients with sepsis who were evaluated by the rapid response team.
Horberg 2021 [16]	Retrospective cohort study; USA; 2013 to 2018.	4549 with fluid and electrolyte disorder or altered mental status, 26 sepsis.	56.1	43.1%	Patients with ED treat-and-release encounters with diagnosis of fluid and electrolyte disorder and altered mental status who were admitted with sepsis within 31 days.
Husabo 2020 [21]	Retrospective cohort study; Norway; 2015 to 2017.	1559 infections	67.0	51.3%	Patients were seen in 24 EDs in Norway with infection and at least 2 systemic inflammatory response syndrome criteria.
Jing 2025 [22]	Retrospective cohort study; China; 2021 to 2024.	183 sepsis	65.7	59.6%	Patients were adults with sepsis treated at the First People’s Hospital of Linping.
Kaal 2022 [23]	Retrospective cohort study; Netherlands; 2019 to 2020.	1477 suspected infections	Adults	-	Patients were adults admitted after presentation at the ED with blood cultures taken.
Kathi 2025 [24]	Retrospective cohort study; Hungary; 2023.	199 total, 78 sepsis	Adults	-	Patients were adults with procalcitonin levels >0.1 ng/mL.
Kirkpatrick 2013 [25]	Retrospective cohort study; USA; 2008 to 2010.	35 sepsis	66	45%	Adult patients with severe sepsis who were not admitted to the ICU from the emergency department.
Martin 2019 [26]	Retrospective cohort study; USA; Unclear.	18 sepsis	-	-	Patients were ICU upgrades with a subgroup with sepsis.
Michelson 2023 [11]	Secondary analysis of retrospective cross-sectional study; USA; 2010 to 2019.	646 sepsis	Under age 21 years	52.8%	Patients were children with sepsis who visited one of 5 pediatric EDs.
Miller 2019 [27]	Retrospective cohort study; USA; 2017.	500 sepsis	-	-	Patients with sepsis or septic shock in Michigan Medicine.
Morr 2017 [28]	Retrospective cohort study; Germany; Unclear.	110 total, 54 sepsis	Median 59 (full cohort)	58.2% (full cohort)	Adult patients with sepsis whp presented to a university hospital ED.
Nassery 2021 [29]	Retrospective cohort study; USA; 2013 to 2018.	3468 sepsis	Adults	-	Patients were adults in the Kaiser Permanente Mid-Atlantic States database with sepsis hospitalization.
Nguyen-Luu 2024 [14]	Retrospective cohort study; USA; 2023.	123 sepsis	Adults	-	Patients were adults with severe sepsis or septic shock in the Geisinger Community Medical Center.
Pandey 2025 [30]	Prospective cohort study; USA; 2020 to 2022.	275 sepsis, 2209 no sepsis	-	57.5%	Patients at an urban academic hospital who were critically ill medical patients presenting to the ED resuscitation area.
Pino 2019 [31]	Retrospective cohort study; USA; 2015 to 2018.	727 sepsis, 246 heart failure	75	54.7%	Patients with a diagnosis of sepsis and heart failure admitted to a tertiary cardiovascular center.
Prasad 2020 [32]	Retrospective cohort study; USA; 2012 to 2016.	16,124 sepsis	-	52.8%	Patients were adults with blood cultures, intravenous antibiotics, and sepsis within 72 h of presentation to an ED.
Ramanathan 2013 [33]	Retrospective cohort study; USA; 2012.	36 sepsis	-	-	Patients with surgical mortalities with concurrent sepsis among hospitalized patients.
Sawicki 2025 [34]	Retrospective cohort study; USA; 2004 to 2023.	184,157 sepsis	Children	-	Patients hospitalized for sepsis in a pediatric health information system-participating hospital.
Scott 2018 [35]	Retrospective cohort study; USA; 2014 to 2015.	1094 sepsis	Children	48.7%	Patients were children with sepsis in a tertiary and community ED.
Souganidis 2022 [36]	Retrospective cohort study; USA; 2014 to 2017.	972 sepsis	Children	57.4%	Patients with sepsis in a tertiary care children’s hospital ED.
Struble 2024 [37]	Retrospective cohort study; USA; 2016 to 2019.	649,756 sepsis	-	-	Patients had sepsis hospitalization in the Merative MarketScan administrative claims data.
Van der Wekken 2016 [38]	Retrospective cohort study; Netherlands; 2012.	131 sepsis	65.6 (full cohort)	56.6%	Patients with sepsis who were treated by emergency medical services staff.

ED = emergency department.

**Table 2:** Study quality assessment.

Study ID	Representativeness of a Population with Sepsis	Reliability of Diagnostic Error Evaluation	Evaluation of Outcome Was Not Present at Start of Study	Use of Adjustments if Appropriate	Reliability of Outcome if Applicable	Adequacy of Follow-Up Period	Adequacy of Follow-Up
Abe 2019 [13]	Yes, adults with suspected infection.	Yes, misdiagnosis of site of infection was defined as discrepancy between initial and final diagnosis or infection that occurred in unidentified site.	Yes, mortality outcome.	Yes, adjusted model and propensity-matched models.	Yes, in-hospital mortality.	Yes, in-hospital outcomes and 28-day mortality.	No, 86 (8.1%) patients were excluded for not having an infection or missing data from the infection site.
Barker 2023 [19]	Yes, adults admitted to hospital.	Yes, delayed diagnosis based on absence of sepsis in provisional diagnosis.	Not applicable, no outcomes.	Not applicable, no outcomes.	Not applicable, no outcomes.	Not applicable, no outcomes.	Not applicable, no outcomes.
Bosetti 2024 [12]	Yes, adults seen in the emergency department with sepsis.	Yes, recognized and unrecognized sepsis.	Yes, outcome on discharge.	No, not adjusted.	Yes, in-hospital mortality.	Yes, in-hospital outcome.	Yes, 1745 (4.1%) excluded, which includes a subset for missing data.
Cifra 2021 [15]	Yes, children with severe sepsis/septic shock.	Yes, patients with potentially missed diagnosis of sepsis with an ED within 7 days before admission.	Not applicable, no outcomes.	Not applicable, no outcomes.	Not applicable, no outcomes.	Not applicable, no outcomes.	Not applicable, no outcomes.
Hamdon 2019 [20]	Yes, patients with sepsis.	Yes, missed diagnosis of sepsis.	Not applicable, no outcomes.	Not applicable, no outcomes.	Not applicable, no outcomes.	Not applicable, no outcomes.	Not applicable, no outcomes.
Horberg 2021 [16]	No, patients with fluid and electrolyte disorder and altered mental status diagnosis in ED.	Yes, readmission with a diagnosis of sepsis within 31 days.	Not applicable, no outcomes.	Not applicable, no outcomes.	Not applicable, no outcomes.	Not applicable, no outcomes.	Not applicable, no outcomes.
Husabo 2020 [21]	Yes, patients with sepsis.	Yes, not triaged within 15 min and examination not in accordance with priority, lactate not measured within 1 h and inadequate observation.	Yes, delay in antibiotic treatment and 30-day mortality.	Yes, adjusted for organ failure, age, comorbidity, and time to admission.	Yes, likely reliable based on clinical records.	Yes, in-hospital antibiotic use and 30-day mortality.	No, 114 (7.3%) incomplete data for antibiotics.
Jing 2025 [22]	Yes, patients with sepsis-3 diagnostic criteria.	Yes, early recognition identified as sepsis within 6 h of admission.	Yes, in-hospital mortality.	No, only unadjusted results for early recognition and mortality.	Yes, likely reliable based on clinical records.	Yes, in-hospital mortality.	No, 92/275 (33.5%) excluded, which includes missing data.
Kaal 2022 [23]	Yes, patients with suspected bacteremia.	Yes, discrepancy between the suspected site of infection at admission and discharge.	Yes, 30-day mortality, intensive care unit admission, and length of stay.	Yes, adjusted for age, Charlson comorbidity index, quick Sepsis-Related Organ Failure Assessment score, and site of infection at discharge.	Yes, likely reliable based on clinical records.	Yes, 30-day mortality.	Unclear, missing data not defined.
Kathi 2025 [24]	Yes, patients with elevated procalcitonin >0.1 ng/mL.	Yes, misdiagnosis was defined by not meeting the SIRS criteria.	Not applicable, no outcomes.	Not applicable, no outcomes.	Not applicable, no outcomes.	Not applicable, no outcomes.	Not applicable, no outcomes.
Kirkpatrick 2013 [25]	Yes, patients with a diagnosis of sepsis.	Yes, diagnostic delay defined by meeting severe sepsis criteria but no physician notation of severe sepsis or antibiotic order within 6 h of severe sepsis onset.	Not applicable, no outcomes.	Not applicable, no outcomes.	Not applicable, no outcomes.	Not applicable, no outcomes.	Not applicable, no outcomes.
Martin 2019 [26]	Yes, patients with sepsis.	Yes, patients with misdiagnosis related to sepsis, either failure to recognize sepsis or misattribution of the source of sepsis.	Not applicable, no outcomes.	Not applicable, no outcomes.	Not applicable, no outcomes.	Not applicable, no outcomes.	Not applicable, no outcomes.
Michelson 2023 [11]	Yes, patients with sepsis.	Yes, delayed diagnosis based on manual expert case review.	Not applicable, no outcomes.	Not applicable, no outcomes.	Not applicable, no outcomes.	Not applicable, no outcomes.	Not applicable, no outcomes.
Miller 2019 [27]	Yes, patients with sepsis.	Yes, misdiagnosed patients had an outpatient visit within 1 day of admission.	Not applicable, no outcomes.	Not applicable, no outcomes.	Not applicable, no outcomes.	Not applicable, no outcomes.	Not applicable, no outcomes.
Morr 2017 [28]	Yes, patients with sepsis.	Yes, recognized and unrecognized sepsis.	Yes, ICU admission for mortality.	No, only unadjusted results for in-hospital mortality and ICU admission.	Yes, likely reliable based on clinical records.	Yes, in-hospital mortality and ICU admission.	No, 15 patients with incomplete data collection or readmission.
Nassery 2021 [29]	Yes, patients with sepsis.	Yes, patients with an ED visit 1 month before.	Not applicable, no outcomes.	Not applicable, no outcomes.	Not applicable, no outcomes.	Not applicable, no outcomes.	Not applicable, no outcomes.
Nguyen-Luu 2024 [14]	Yes, patients with sepsis.	Yes, false negatives (missed sepsis) and false positives.	Not applicable, no outcomes.	Not applicable, no outcomes.	Not applicable, no outcomes.	Not applicable, no outcomes.	Not applicable, no outcomes.
Pandey 2025 [30]	Yes, patients with sepsis.	Yes, patients with missed sepsis and overdiagnosis of sepsis.	Yes, in-hospital mortality.	No, only unadjusted results for in-hospital mortality.	Yes, likely reliable based on clinical records.	Yes, in-hospital mortality.	No, 62 patients excluded for no initial visual analog scale score and ineligible after review.
Pino 2019 [31]	Yes, patients with sepsis.	No, patients with diagnosis of acute decompensated heart failure.	Not applicable, no outcomes.	Not applicable, no outcomes.	Not applicable, no outcomes.	Not applicable, no outcomes.	Yes, 1 patient not accounted for, as 974 patients but results only report 973.
Prasad 2020 [32]	Yes, patients with sepsis.	Yes, sepsis recognition based on SIRS and SOFA criteria.	Yes, in-hospital mortality.	No, only unadjusted results for in-hospital mortality.	Yes, likely reliable based on clinical records.	Yes, in-hospital mortality.	No, 1089 (6.2%) excluded for missing data.
Ramanathan 2013 [33]	No, surgical mortality with sepsis.	Yes, misdiagnosis based on physician reviewer.	Not applicable, no outcomes.	Not applicable, no outcomes.	Not applicable, no outcomes.	Not applicable, no outcomes.	Not applicable, no outcomes.
Sawicki 2025 [34]	Yes, patients with sepsis in children.	Yes, patients with one emergency department visit in the 30 days prior to sepsis hospitalization.	Not applicable, no outcomes.	Not applicable, no outcomes.	Not applicable, no outcomes.	Not applicable, no outcomes.	Not applicable, no outcomes.
Scott 2018 [35]	Yes, patients with sepsis in children.	Yes, missed sepsis based on the absence of a sepsis timestamp in records.	Yes, 30-day mortality.	No, only unadjusted results for 30-day mortality.	Yes, likely reliable based on clinical records.	Yes, 30-day mortality.	Unclear, missing data not defined.
Souganidis 2022 [36]	Yes, patients with sepsis in children.	Yes, missed sepsis defined by care without the use of the ED sepsis pathway/order set.	Yes, intravenous antibiotics within 90 min of ED triage.	No, unadjusted results only.	Yes, likely reliable based on clinical records.	Yes, in-hospital antibiotics.	Unclear, missing data not defined.
Struble 2024 [37]	Yes, patients with sepsis.	Yes, diagnostic delay.	Not applicable, no outcomes.	Not applicable, no outcomes.	Not applicable, no outcomes.	Not applicable, no outcomes.	Not applicable, no outcomes.
Van der Wekken 2016 [38]	Yes, patients with sepsis.	Yes, sepsis recognition by emergency medical service staff.	Not applicable, no outcomes.	Not applicable, no outcomes.	Not applicable, no outcomes.	Not applicable, no outcomes.	Not applicable, no outcomes.

ED = emergency department, ICU = intensive care unit, SIRS = systemic inflammatory response syndrome, SOFA = sequential organ failure assessment.

**Table 3:** Definition and rates of diagnostic errors in sepsis.

Study ID	Definition of Diagnostic Error in Adults	Rate of Diagnostic Error
Abe 2019 [13]	Misdiagnosis of site of infection was defined as a discrepancy between initial and final diagnoses or infection that occurred in an unidentified site.	Misdiagnosis of site of infection among hospital admissions: 113/974 (11.6%).
Barker 2023 [19]	Delayed diagnosis of sepsis defined by the absence of sepsis in the provisional diagnosis.	Delayed diagnosis among hospital admissions with sepsis: 43/59 (72.9%).
Bosetti 2024 [12]	Recognized sepsis was defined as patients with a high-priority code, while unrecognized sepsis was defined as patients with a low-priority code.	Unrecognized sepsis in the emergency department: 3794/11,508 (33.0%).
Hamdon 2019 [20]	Missed sepsis defined by other diagnosis by the rapid response team.	Missed sepsis among rapid response team assessments: 16/22 (72.7%).
Horberg 2021 [16]	Misdiagnosis of sepsis based on sepsis hospital admission within 31 days of ED treat-and-release encounter for fluid and electrolyte disorder or altered mental status.	Misdiagnosis based on readmission with sepsis after ED encounter with fluid and electrolyte disorder or altered mental status: 26/4549 (0.57%).
Husabo 2020 [21]	Delay to diagnosis defined by delay to antibiotic treatment based on: not being triaged within 15 min, examination by physician not in accordance with priority, lactate not measured within 1 h, and inadequate observation regimen.	Not being triaged within 15 min: 27.1% (422/1559). Examination by physician not in accordance with priority: 55.1% (859/1559). Lactate not measured within 1 h: 51.4% (801/1559). Inadequate observation regimen: 55.6% (867/1524). Delay to antibiotic treatment beyond 1 year: 74.6% (1078/1445).
Jing 2025 [22]	Early recognition of sepsis defined by identification of sepsis within 6 h of admission.	Delayed recognition of sepsis after 6 h: 47/183 (25.7%).
Kaal 2022 [23]	Misdiagnosis was defined as a discrepancy between the suspected site of infection at hospital admission and the final diagnosis at discharge.	Misdiagnosis rate of site of infection: 171/1477 (11.6%).
Kathi 2025 [24]	Misdiagnosis was defined when patients did not meet SIRS criteria.	Misdiagnosis rate of patients with sepsis who did not meet the sepsis criteria: 43/78 (55.1%). Misdiagnosis rate for patients who had another diagnosis and met the sepsis criteria: 46/121 (38.0%).
Kirkpatrick 2013 [25]	Diagnostic delay defined as meeting severe sepsis criteria but physician notation of a severe sepsis diagnosis and/or antibiotic orders were not documented within 6 or 12 h of severe sepsis onset.	Diagnostic delay of greater than 6 h: 12/35 (34.3%). Diagnostic delay of greater than 12 h in recognition or treatment of severe sepsis: 7/35 (20.0%).
Martin 2019 [26]	Misdiagnosis of sepsis was either failure to recognize sepsis or misattribution of the source of sepsis.	Among ICU upgrades, the misdiagnosis rate was 14/18 (77.8%).
Miller 2019 [27]	Misdiagnosis defined by an outpatient visit within 1 day of admission with sepsis.	Misdiagnosis based on outpatient visit 1 day before admission: 49/500 (9.8%).
Morr 2017 [28]	Sepsis recognition defined by emergency department doctor documentation compared to classification by study team.	Sepsis not correctly used in 32/54 patients (59.3%).
Nassery 2021 [29]	Sepsis misdiagnosis based on 1-month ED visit prior to sepsis hospitalization.	Misdiagnosis based on 1-month ED visit prior to sepsis hospitalization: 766/3468 (22.1%).
Nguyen-Luu 2024 [14]	Sepsis misdiagnosis based on false positives and false negatives.	Error rate for sepsis: 67/148 (45.3%). False positive rate for sepsis: 8/73 (11.0%). False negative rate for sepsis: 59/79 (78.7%).
Pandey 2025 [30]	Missed sepsis defined by the absence of explicit sepsis diagnosis on arrival. Sepsis overdiagnosis defined by false diagnosis of sepsis on arrival.	Missed sepsis within 60 min: 48/275 (17.5%). Persistent missed sepsis beyond 60 min: 23/275 (8.4%). Sepsis overdiagnosis: 333/2209 (15.1%).
Pino 2019 [31]	Misdiagnosis of sepsis in patients with acute decompensated heart failure.	Misdiagnosis of sepsis in heart failure patients: 246/974 (25.3%).
Prasad 2020 [32]	Sepsis recognition based on SIRS-first or SOFA-first, and the difference in timing was a median of 26 min and 113 min, respectively.	Sepsis recognition: SIRS first (median 26 min): 9087/16,124 (54.7%). SOFA first (median 113 min): 7037/16,124 (42.4%).
Ramanathan 2013 [33]	Misdiagnosis of sepsis based on physician review among surgical mortalities.	Misdiagnosis of sepsis among surgical mortalities: 3/36 (8.3%).
Van der Wekken 2016 [38]	Failure to recognize sepsis by emergency medical services staff.	Failure to recognize sepsis by emergency medical services staff: 113/131 (86.2%).
**Study ID**	**Definition of Diagnostic Error in Children**	**Rate of Diagnostic Error**
Cifra 2021 [15]	Missed sepsis defined by an emergency department visit within 7 days of admission with sepsis in children.	Missed sepsis among children with sepsis admissions in hospital: 158/1945 (8.1%).
Michelson 2023 [11]	Delay in diagnosis defined by sepsis in a child being present at the initial encounter based on manual expert case review.	Delay in diagnosis of sepsis in a child: 109/646 (16.9%).
Sawicki 2025 [34]	Missed sepsis based on at least one ED treat-and-release visit in the preceding 30 days prior to child sepsis admission.	Missed diagnosis of sepsis in children: 15,331/184,157 (8.3%).
Scott 2018 [35]	Missed sepsis were cases of sepsis in the children’s registry that did not have a sepsis time stamp in the electronic health record.	Missed sepsis in children: 32/1094 (2.9%).
Sougandis 2022 [36]	Missed sepsis defined by the absence of an ED sepsis pathway/order set in a child.	Missed sepsis in children: 53/972 (5.5%).

ED = emergency department, ICU = intensive care unit, SIRS = systemic inflammatory response syndrome, SOFA = sequential organ failure assessment.

**Table 4:** Outcomes associated with diagnostic errors in sepsis.

Study ID	Outcomes in Studies of Adults
Abe 2019 [13]	In-hospital mortality with misdiagnosis of site of infection vs. correct site: Crude results 28/113 (24.8%) vs. 118/861 (13.7%), *p* < 0.01. Adjusted odds ratio with generalized estimation equation aOR 2.66 95% CI 1.45–4.89. Propensity-matched model aOR 3.03 95% CI 1.24–7.38. Misdiagnosis of site of infection vs. correct site: 28-day mortality 20/97 (20.6%) vs. 101/756 (13.4%), *p* = 0.05. Length of stay: median 15 (8–26) days vs. 14 (8–28) days, *p* = 0.59.
Bosetti 2024 [12]	In-hospital mortality for unrecognized and recognized sepsis: Crude results 86/3794 (2.3%) vs. 805/7714 (10.4%), *p* < 0.001. Median length of stay in ED for unrecognized and recognized sepsis: 16.8 (5.3–33.9) vs. 14.3 (4.7–28.3) days, *p* < 0.001.
Husabo 2020 [21]	Multiple linear regression for delay in antibiotics with: Not being triaged within 15 min: b 025.8 95% CI 3.8 to 47.8. Examination by physician not in accordance with priority: b 38.09 95% CI 16.1 to 59.8. Lactate not measured within 1 h: b 71.4 95% CI 56.0 to 86.8. Inadequate observation regimen: 23.9 95% CI 10.5 to 37.3. U-shaped relationship between 30-day all-cause mortality and time to antibiotics with 10% mortality for 0 h, 5% for 2 to 7 h, and 7% beyond 8 h.
Jing 2025 [22]	Early vs. late recognition: SOFA score: 6.5 ± 1.3 vs. 9.2 ± 1.6, *p* < 0.001. Use of anti-infective treatment: 129/136 (94.9%) vs. 41/47 (87.2%), *p* = 0.155. Duration in hospital: 12.5 ± 1.2 vs. 14.6 ± 2.5, *p* < 0.001. Mortality rate: 14/136 (10.3%) vs. 12/47 (25.5%), *p* = 0.010.
Kaal 2022 [23]	Misdiagnosis and adjusted odds of: 30-day mortality: aOR 0.8 95% CI 0.3–1.9, *p* = 0.60. ICU admission: aOR 1.3 95% CI 0.6–3.0, *p* = 0.54. Duration of admission: adjusted effect size 1.1 95% CI 0.9–1.3, *p* = 0.41. Receiving broad-spectrum antibiotic therapy: aOR 4.0 95% CI 1.8–8.8, *p* < 0.001.
Morr 2017 [28]	Unrecognized and recognized sepsis and outcomes: Appropriate antibiotics: 32/32 (100%) vs. 21/22 (96%), *p* = 0.42. Mortality or ICU admission: 4/32 (13%) vs. 4/22 (18%), *p* = 0.34.
Nassery 2021 [29]	Patients with fluid electrolyte disorders (observed-to-expected ratio 2.66) and altered mental status (observed-to-expected ratio 2.82) were most likely to have downstream sepsis admission.
Nguyen-Luu 2024 [14]	Mortality rate according to sepsis group: True positive: 6/65 (9.2%). True negative: 10.17% (n = 16). False positive: 3/8 (37.5%). False negative: 6.87% (n = 4).
Pandey 2025 [30]	Missed sepsis and outcomes: Antibiotics within 60 min: 31/48 (64.6%) vs. 164/227 (72.2%). Mortality: 7/48 (14.6%) vs. 40/227 (17.6%). Persistent missed sepsis and outcomes: Antibiotics within 60 min: 12/23 (52.2%) vs. 183/252 (72.6%). Mortality: 2/23 (8.7%) vs. 45/252 (17.9%).
Prasad 2020 [32]	Sepsis recognition and outcomes for SIRS first (median 26 min) vs. SOFA first (median 113 min): In-hospital mortality: 579/9087 (6.4%) vs. 479/7037 (6.8%), *p* = 0.27. ICU length of stay: 2.8 [1.7 to 5.1] vs. 3.0 [1.8 to 5.1], *p* = 0.14.
**Study ID**	**Outcomes in Studies of Children**
Scott 2018 [35]	Identified vs. missed sepsis among children and outcomes: ICU admission within 24 h: 28/32 (87.5%) vs. 324/1062 (30.5%). 30-day mortality: 0/32 (0%) vs. 15/1062 (1.4%).
Sougandis 2022 [36]	Missed and identified sepsis and intravenous antibiotics within 90 min of emergency department triage: 15/53 (28.3%) vs. 493/919 (53.6%), relative risk 0.4 95% CI 0.3–0.7.

ED = emergency department, aOR = adjusted odds ratio, CI = confidence interval, ICU = intensive care unit, SIRS = systemic inflammatory response syndrome, SOFA = sequential organ failure assessment.

## Data Availability

Data used for this study is derived from published data. The data is available from the authors of the original studies.

## Supplementary Materials

The following supporting information can be downloaded at: https://www.mdpi.com/article/10.3390/jcm15208152/s1.

## Abbreviations

The following abbreviations are used in this manuscript:

SOFA	Sequential Organ Failure Score
WHO	World Health Organization
PRISMA	Preferred Reporting Items for Systematic Reviews and Meta-Analyses
ED	emergency department
ICD	international classification of disease
SIRS	systemic inflammatory response syndrome
